# Transfusion‐Related Acute Lung Injury: from Mechanistic Insights to Therapeutic Strategies

**DOI:** 10.1002/advs.202413364

**Published:** 2025-01-21

**Authors:** Xiaobin Fang, Chunheng Mo, Ling Zheng, Fei Gao, Fu‐Shan Xue, Xiaochun Zheng

**Affiliations:** ^1^ Department of Anesthesiology/Critical Care Medicine Fuzhou University Affiliated Provincial Hospital School of Medicine Fuzhou University Shengli Clinical Medical College of Fujian Medical University Fujian Provincial Key Laboratory of Critical Care Medicine Fujian Provincial Hospital Fuzhou Fujian 350001 China; ^2^ Key Laboratory of Birth Defects and Related Diseases of Women and Children of MOE State Key Laboratory of Biotherapy West China Second University Hospital Sichuan University Chengdu 610041 China; ^3^ Department of Anesthesiology Fujian Provincial Hospital Shengli Clinical Medical College of Fujian Medical University & Fujian Emergency Medical Center Fujian Provincial Key Laboratory of Emergency Medicine Fujian Provincial Key Laboratory of Critical Medicine Fujian Provincial Co‐constructed Laboratory of “Belt and Road,” Fuzhou Fujian China

**Keywords:** immune response, pathogenesis, signaling pathways, therapeutic strategies, transfusion‐related acute lung injury (TRALI)

## Abstract

Transfusion‐related acute lung injury (TRALI) is a potentially lethal complication of blood transfusions, characterized by the rapid onset of pulmonary edema and hypoxemia within six hours post‐transfusion. As one of the primary causes of transfusion‐related mortality, TRALI carries a significant mortality rate of 6–12%. However, effective treatment strategies for TRALI are currently lacking, underscoring the urgent need for a comprehensive and in‐depth understanding of its pathogenesis. This comprehensive review provides an updated and detailed analysis of the current landscape of TRALI, including its clinical presentation, pathogenetic hypotheses, animal models, cellular mechanisms, signaling pathways, and potential therapeutic targets. By highlighting the critical roles of these pathways and therapies, this review offers valuable insights to inform the development of preventative and therapeutic strategies and to guide future research efforts aimed at addressing this life‐threatening condition.

## Graphical abstract

This review systematically presents an updated and comprehensive overview of transfusion‐related acute lung injury (TRALI), a serious complication of blood transfusions. It explores TRALI's clinical features, potential causes, and immune mechanisms. Key insights include the roles of signaling pathways and possible therapies, with a focus on prevention strategies and future research directions.

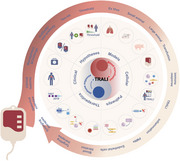

## 1 Introduction

Transfusion‐related acute lung injury (TRALI), a life‐threatening complication that can occur within six hours of blood transfusion, is characterized by sudden‐onset hypoxemic respiratory failure without cardiogenic pulmonary edema.^[^ ^1^ ^]^ Historically considered rare,^[^ ^2^ ^]^ perceptions of TRALI have changed significantly over the past two decades. An international consensus definition established over a decade ago sparked intense research,^[^ ^3^ ^]^ revealing a higher incidence in specific patient populations. A recently emerged new definition reflects advances in our understanding of TRALI pathophysiology, contributing to improved diagnostic accuracy.^[^ ^1b^ ^]^ These evolving definitions have spurred advancements in TRALI‐related clinical and mechanistic studies, facilitating the development of treatment strategies.

Since its first report in 1951,^[^ ^4^ ^]^ TRALI has evolved from being recognized as an allergic reaction to a major transfusion complication in the 1980s.^[^ ^2^, ^5^ ^]^ By the 1990s, TRALI had become a leading cause of transfusion‐related mortality.^[^ ^6^ ^]^ The international consensus refined the definitions and diagnostic criteria of TRALI from the 2004 Canadian Consensus^[^ ^3^ ^]^ to the 2019 update,^[^ ^7^ ^]^ enhancing our understanding of its clinical impact. The lack of diagnostic markers and targeted therapies for TRALI necessitates research into its pathogenesis, leading to various hypotheses, including antibody‐^[^ ^8^ ^]^ and non‐antibody‐mediated mechanisms^[^ ^9^ ^]^ as well as the “two‐hit”^[^ ^10^ ^]^ and threshold models.^[^ ^11^ ^]^ Studies using animal models have highlighted the crucial role of the immune system in TRALI pathogenesis.^[^ ^12^ ^]^ Advancements in mechanistic research have led to TRALI prevention strategies, ranging from selective use of donor blood products^[^ ^13^ ^]^ to targeting specific blood product components^[^ ^14^ ^]^ and developing specific signaling pathway‐targeting therapies.^[^ ^15^ ^]^ Previous reviews have provided important insights into TRALI's clinical,^[^ ^1a^ ^]^ mechanistic,^[^ ^16^ ^]^ and therapeutic aspects.^[^ ^17^ ^]^ However, no systematic review integrates these insights for a comprehensive understanding.

In this comprehensive review, we aimed to consolidate knowledge on the clinical features, mechanism research, and therapeutic strategies of TRALI, enhancing our understanding of its pathophysiology and informing better preventive and therapeutic approaches. We begin with an overview of its clinical presentation, followed by an exploration of the underlying hypotheses and mechanisms. Next, we discuss relevant animal models and conclude with a review of the pathways involved, along with current and emerging therapeutic approaches. Finally, we provide essential insights into these life‐threatening transfusion reactions for future studies to improve patient care and outcomes.

## 2 Clinical Overview

### 2.1 Historical Overview and Epidemiology

**Figure 1:**
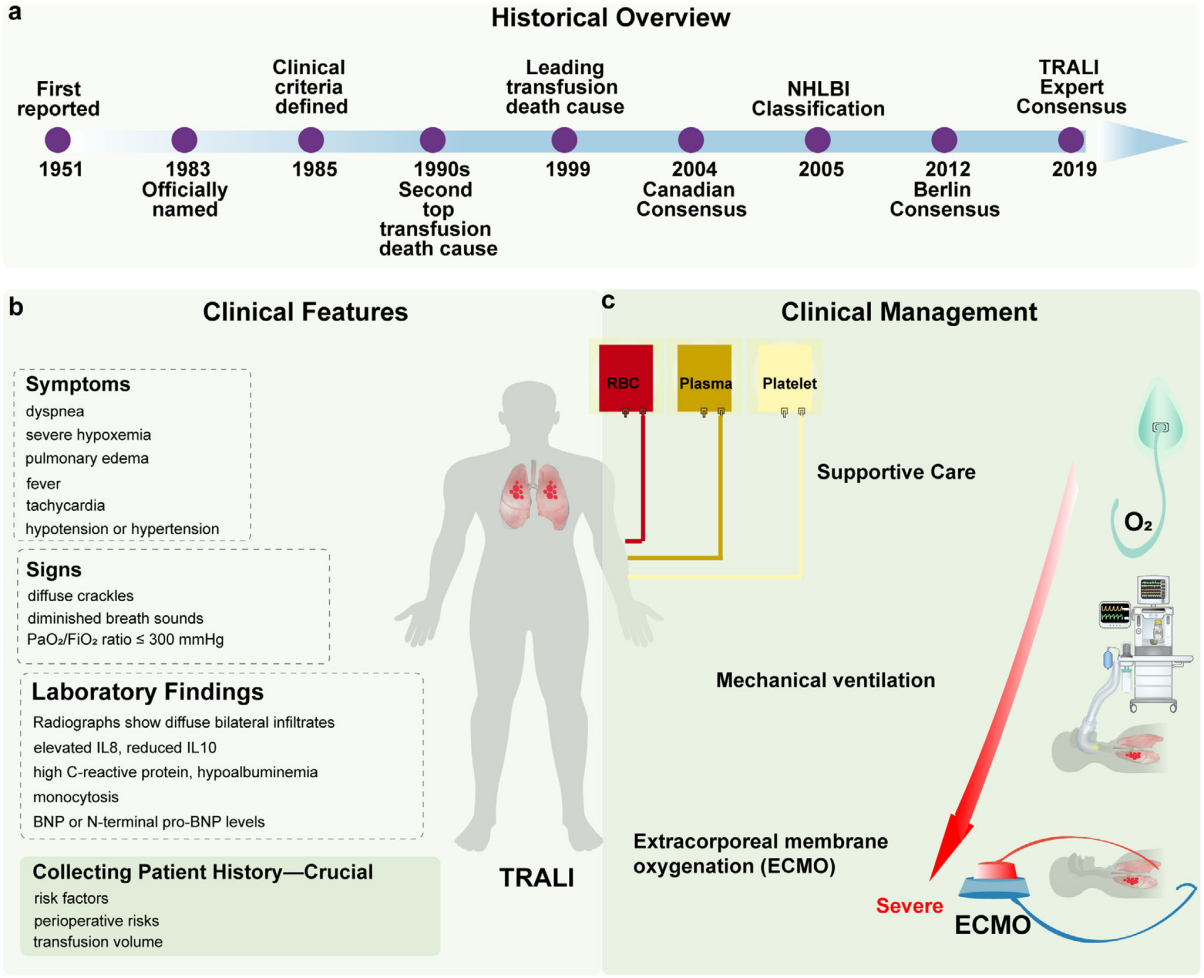
Clinical overview of TRALI. TRALI has high mortality and incidence in critically ill patients, with a lack of specific diagnostic markers and targeted therapies. The accurate definition drives research advancements. a) Historical overview of TRALI; b) Clinical features of TRALI; c) Clinical management of TRALI.

TRALI was first described by Barnard in 1951 as a non‐cardiogenic post‐transfusion pulmonary edema attributed to an allergic reaction.^[^ ^4^ ^]^ Subsequent research associated TRALI with donor anti‐leukocyte antibodies and human leukocyte antigen (HLA)‐ incompatibility.^[^ ^18^ ^]^ The official name “transfusion‐related acute lung injury” was proposed by Popovsky et al. in 1983.^[^ ^2^ ^]^ By the 1990s, TRALI was the second leading cause of transfusion‐related deaths (15%)^[^ ^19^ ^]^ and became the top cause by 1999, according to the Serious Hazards of Transfusion (SHOT) report.^[^ ^6^ ^]^ Diagnostic criteria have evolved from the 1985 definition^[^ ^5^ ^]^ to the 2004 Canadian Consensus categorization of “possible” and “definite” TRALI,^[^ ^3^ ^]^ the 2005 National Heart, Lung, and Blood Institute (NHLBI) inclusion of acute lung injury (ALI) risk factors,^[^ ^20^ ^]^ and the 2019 update distinguishing Type I and Type II TRALI.^[^ ^1b^ ^]^ These updates improved diagnostic precision and guided research (**Figure** **1**a).

TRALI incidence varies widely, estimated at 0.08–15.1% per patient and 0.01–1.12% per product, and up to 15% in critically ill patients.^[^ ^1^, ^21^ ^]^ Pediatric perioperative TRALI rates are lower (0.1–2%), but critically ill pediatric patients may experience rates as high as 29.4%.^[^ ^22^ ^]^ Patients in ICUs are at the highest risk, with mortality rates ranging from 21–90%.^[^ ^21^, ^23^ ^]^ The SHOT report estimates that TRALI accounts for approximately 21% of transfusion‐related deaths.^[^ ^24^ ^]^

### 2.2 Clinical Features and Diagnosis

TRALI presents as rapid ALI onset within 6 h post‐transfusion, with non‐specific symptoms such as severe hypoxemia, pulmonary edema, dyspnea, tachycardia, fever, and hypotension without circulatory overload (Figure 1b).^[^ ^20^, ^25^ ^]^ Clinical findings include diffuse crackles, diminished breath sounds, and a PaO_2_/FiO_2_ ratio below 300 mmHg.^[^ ^26^ ^]^ Radiological imaging shows bilateral pulmonary infiltrates consistent with non‐cardiogenic pulmonary edema,^[^ ^1^, ^23^ ^]^ and laboratory markers like elevated interleukin 8 (IL8) and C‐reactive protein, reduced IL10, and hypoalbuminemia offer limited diagnostic specificity.^[^ ^27^ ^]^ The 2019 consensus definitions refined diagnostic criteria, distinguishing Type I TRALI (no ARDS risk factors) from Type II (with ARDS risk factors or mild ARDS),^[^ ^1b^ ^]^ improving diagnostic accuracy.

### 2.3 Management and Challenges

TRALI poses a critical risk to transfusion safety, especially in severely ill patients, due to its high mortality rate.^[^ ^28^ ^]^ Current management is limited to supportive care and ARDS‐derived therapies, which do not target TRALI‐specific mechanisms.^[^ ^27^, ^29^ ^]^ Emerging treatments, such as platelet depletion, aspirin, and extracorporeal membrane oxygenation, show promise but require further validation (Figure 1c).^[^ ^30^ ^]^ Despite advances in diagnostic criteria, it remains underdiagnosed due to overlapping symptoms with ARDS and other pulmonary conditions.^[^ ^25^, ^31^ ^]^ The lack of specific biomarkers for TRALI remains a major challenge, with diagnosis relying on clinical and radiological findings.^[^ ^32^ ^]^ These limitations highlight the urgent need for innovative diagnostic strategies and targeted therapies.

### 2.4 Pathogenetic Hypotheses

**Figure 2:**
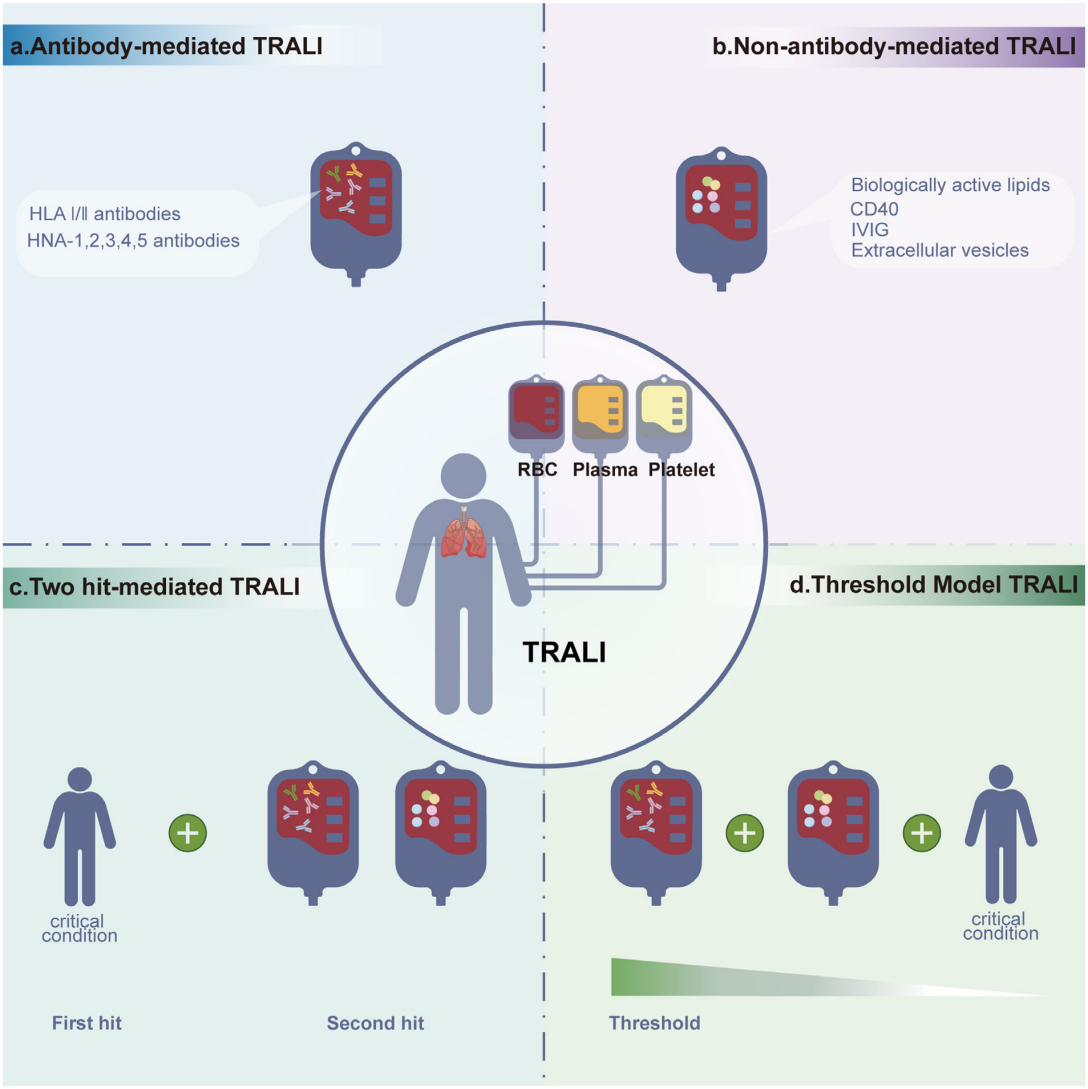
TRALI‐related pathogenetic hypotheses. The threat of TRALI and the lack of diagnostic markers and therapies drive the exploration of its pathogenesis. Four hypotheses have been proposed to explain its pathogenesis. a) antibody‐mediated TRALI; b) non‐antibody‐mediated TRALI; c) Two‐hit‐mediated TRALI; d) threshold models for TRALI.

The high mortality and incidence rates in critically ill patients, combined with the absence of specific diagnostic markers and targeted therapies, drive the need for pathogenesis research. Several hypotheses have been proposed to explain the TRALI‐related pathogenesis, including the antibody‐mediated mechanism, the non‐antibody‐mediated mechanism, and the two‐hit and threshold models, which are crucial for further mechanism research and the development of effective diagnostic and therapeutic strategies (**Figure** **2**).

### 2.5 Antibody‐Mediated TRALI

Predominantly linked to antibodies against HLAs and human neutrophil antigens (HNAs) in the donor blood, antibody‐mediated TRALI accounts for approximately 80% of the cases.^[^ ^8^, ^33^ ^]^ Antibodies targeting HLA Class I and II vary in TRALI severity, with class II being often linked to fatal outcomes.^[^ ^34^ ^]^ HNA‐specific antibody usage frequently leads to severe or fatal TRALI.^[^ ^35^ ^]^ Certain TRALI cases result from recipient antibodies reacting with donor leukocytes, referred to as reverse antibody‐mediated TRALI.^[^ ^36^ ^]^ Comprehensive reviews have already been published of TRALI‐inducing antibodies.^[^ ^37^ ^]^ Based on this mechanism, strategies such as donor gender screening, immunocompatibility tests, or sensitive HLA typing and antibody screenings have been proposed to improve transfusion safety and significantly reduce TRALI incidence.^[^ ^34^, ^38^ ^]^

### 2.6 Non‐Antibody‐Mediated TRALI

To date, antibodies have not been detected in all TRALI cases.^[^ ^10^, ^39^ ^]^ A case report linked pulmonary dysfunction to autologous blood transfusion, suggesting that non‐antibody pathways could also induce TRALI.^[^ ^9c^ ^]^ Most studies suggest that biologically active materials in stored blood products, such as biologically active lipids^[^ ^9a,b^ ^]^ and a cluster of differentiation 40 (CD40),^[^ ^40^ ^]^ could also trigger TRALI. Intravenous immunoglobulin (IVIG)‐related TRALI is another rare, but significant, cause of transfusion‐related mortality, occurring via an antibody‐independent mechanism.^[^ ^41^ ^]^ Recent technological advances revealed extracellular vesicles in blood products, potentially fulfilling immune regulatory roles and contributing to TRALI.^[^ ^14^, ^42^ ^]^ Therefore, further studies targeting these pathogenic agents might effectively prevent TRALI and support its treatment.

### 2.7 “Two‐Hit” Model

Initially proposed by Silliman et al. in 1997, the “two‐hit” model describes TRALI as a result of two sequential clinical insults: a predisposing inflammatory condition followed by transfusion of biologically active substances.^[^ ^9^, ^43^ ^]^ Clinical studies demonstrated that higher PMN‐priming activity in patients with TRALI supports the “two‐hit” hypothesis.^[^ ^9a^ ^]^ This model has evolved to include interactions between leukocyte antibodies and other bioactive substances, delineating a cascade of inflammatory events leading to endothelial damage and pulmonary distress.^[^ ^33^, ^37^, ^43^, ^44^ ^]^ The first hit primes the inflammatory environment, thus activating the pulmonary endothelium and promoting leukocyte migration and adhesion. The second hit, via transfused bioactive substances or antibodies, activates these leukocytes to release inflammatory mediators, causing endothelial damage and pulmonary complications.^[^ ^39^, ^45^ ^]^ Based on the “two‐hit” theory, a new three‐step model attempts to provide a more detailed explanation for TRALI, involving a priming step, pulmonary reaction, and effector phase.^[^ ^16^ ^]^

### 2.8 Threshold Model

As an extension of the two‐hit theory, the threshold model postulates that TRALI stems from the accumulation of predisposing factors and transfusion‐related insults that exceed a specific neutrophil activation threshold.^[^ ^11^, ^45^, ^46^ ^]^ TRALI intensity correlates with these accumulated factors, highlighting a lower and higher threshold for mild and severe cases requiring mechanical ventilation, respectively.^[^ ^11^, ^47^ ^]^ This approach emphasizes the multifactorial nature of TRALI and its variable manifestation among individuals, suggesting that a robust second hit alone could precipitate TRALI under certain conditions.^[^ ^17^, ^48^ ^]^

## 3 Animal Models

**Figure 3:**
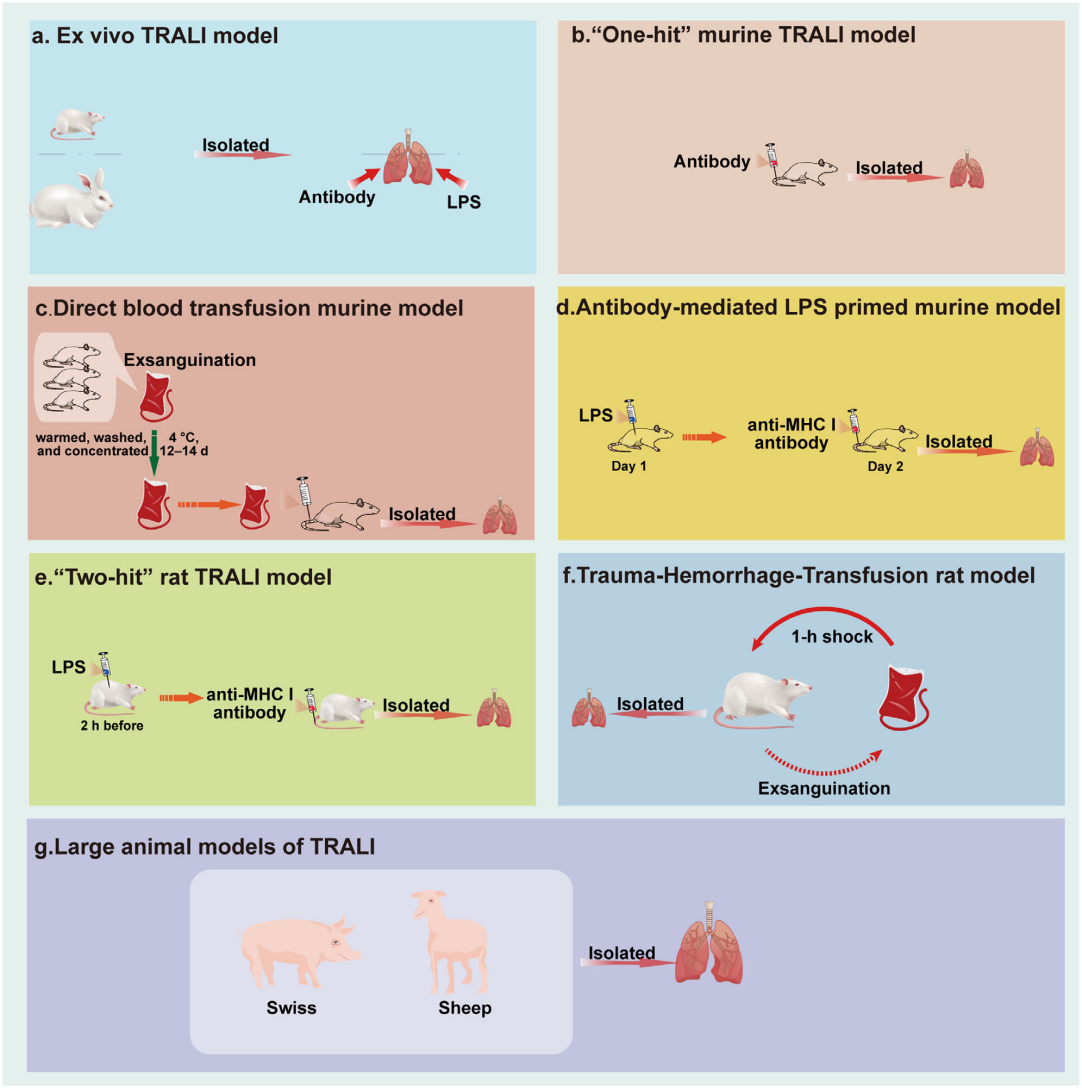
Experimental models for TRALI. While animal models are essential to its mechanism research, they often replicate only parts of a disease mechanism, requiring further optimization. a) Ex vivo TRALI models; b) “One‐hit” murine TRALI model; c) Direct blood transfusion murine model; d) Antibody‐mediated LPS‐primed murine TRALI model; e) “Two‐hit” rat TRALI model; f) Trauma‐hemorrhage‐Transfusion rat TRALI model; g) Large animal models of TRALI.

The hypotheses provide crucial insights into potential underlying mechanisms. However, to validate these hypotheses and further explore cellular mechanisms, robust animal models are essential (**Figure** **3**).

### 3.1 Ex Vivo TRALI Models

Initial TRALI models used ex vivo isolated lungs. Silliman's model involved injecting lipopolysaccharide (LPS) into rats before lung isolation, followed by ventilation and human plasma perfusion to measure lung injury via the lung‐to‐body weight ratio.^[^ ^44d^ ^]^ Seeger's rabbit lung model focuses on antibody‐mediated TRALI.^[^ ^49^ ^]^ However, owing to *ex vivo* setup limitations, such as procedural constraints and the lack of accurate vascular permeability measurements, research has shifted toward more comprehensive in vivo models.^[^ ^50^ ^]^

### 3.2 Small Animal Model of TRALI

#### 3.2.1 “One‐Hit” Murine TRALI Model

The “one‐hit” murine model was introduced at the 2004 TRALI Canada Consensus Conference, established by injecting mice with murine monoclonal alloantibodies (34‐1‐2s) targeting H‐2 class I antigens (analogous to human HLA).^[^ ^20^, ^51^ ^]^ The model animals exhibit early respiratory distress, transient hypothermia, capillary leak, and pulmonary edema, notably affecting male mice only.^[^ ^52^ ^]^

#### 3.2.2 Direct Blood Transfusion Murine Model

This model is adapted for studies focusing on how transfused red blood cells (RBCs) affect the lungs. Murine RBCs are isolated, stored at 4 °C for 12–14 days, and warmed, washed, and concentrated to a hematocrit of 60–70%. RBCs are injected into recipient mice via the tail vein. Six hours later, mice are euthanized, and lung tissue and plasma are collected.^[^ ^53^ ^]^ The evaluation metrics depend on the specific study.^[^ ^54^ ^]^

#### 3.2.3 Antibody‐Mediated LPS‐Primed Murine TRALI Model

The “two‐hit” murine TRALI model involves an initial intraperitoneal LPS injection, followed by the antibody 34‐1‐2s, inducing moderate pulmonary edema in female mice.^[^ ^55^ ^]^ In male mice, LPS is injected the night before, and anti‐BALB/c plasma is administered the next morning, reducing oxygen saturation.^[^ ^50a^ ^]^ This “two‐hit” model is the most commonly used in recent TRALI animal studies.^[^ ^27^, ^30^, ^56^ ^]^ On the day of the experiment, LPS is injected as the first hit, followed by the antibody 34‐1‐2s, with IgG2α as a control.^[^ ^57^ ^]^ To reduce mortality, anesthesia, and mechanical ventilation are coupled with the second hit.^[^ ^58^ ^]^ The evaluation can include lung extravascular water, vascular permeability (using 125I‐labeled albumin), myeloperoxidase level, cytokine, and lung histology assessments.^[^ ^30^, ^57^ ^]^

Building on the “two‐hit” TRALI model, this version involves CD4+ T cell depletion. Eighteen hours prior to TRALI induction, mice receive LPS intraperitoneally, followed by monoclonal antibody GK1.5 administration to deplete CD4+ T cells. TRALI is induced on the day of the experiment using antibodies 34‐1‐2s and AF6‐88.5.5.3.^[^ ^59^ ^]^

#### 3.2.4 “Two‐Hit” Rat TRALI Model

Presented at the 2006 ASH meeting, this model involves two steps in male rats. Step 1 involves the injection of 2 mg kg^−1^LPS intraperitoneally (using saline as a control) 2 h before Step 2, which corresponds to anesthetizing the rats, cannulating the femoral vessels, and withdrawing 5–10% of the blood volume. Within 30 min, the animals are infused with stored plasma, monoclonal antibodies, anti‐neutrophil serum, or saline, followed by Evans blue dye to assess capillary leakage.^[^ ^60^ ^]^ This model yields lower mortalities than the mouse model, thereby closely mimicking clinical conditions.

#### 3.2.5 Trauma–Hemorrhage–Transfusion Rat TRALI Model

Most TRALI models utilize LPS and antibody transfusion as the first and second hits, respectively. However, this process does not mimic clinical trauma and surgery scenarios. A new model uses hemorrhagic shock and RBC transfusion as the first and second hits, respectively, to better understand TRALI and explore preventive strategies.^[^ ^61^ ^]^ Rats are anesthetized and undergo femoral catheterization, and 30% of the blood volume is withdrawn in 10 min, followed by a 1‐h shock period, and, finally, erythrocyte suspension administration.^[^ ^15^, ^61^ ^]^

### 3.3 Large Animal Model of TRALI

Large animal models, such as sheep, could more closely mimic human TRALI cases. Tung et al. developed a model where female Merino sheep, under anesthesia and ventilation, receive an initial saline or LPS infusion, followed by a second saline or pooled human platelet concentrate and plasma mix infusion, amounting to 10% of the total blood volume.^[^ ^62^ ^]^ Subsequently, Tung et al. validated this model using freshly packed RBCs to minimize TRALI risk, thereby confirming feasibility.^[^ ^63^ ^]^

Finally, a swine TRALI model was developed using low LPS doses and a monoclonal antibody against swine leukocyte antigen class I. Neutrophils are depleted four days prior to using cyclophosphamide. On the day of the experiment, pigs are anesthetized and ventilated. Upon stabilization, the animals are infused with LPS or saline, followed by SLA class I MoAb or control MoAb infusion. Lung injury is then assessed using PaO_2_/FiO_2_, chest X‐ray, and histopathology.^[^ ^64^ ^]^ Finally, another study used pigs to develop an erythrocyte transfusion model, assessing lung function, tidal volume, and inflammatory markers.^[^ ^65^ ^]^

## 4 Cellular Mechanisms

**Figure 4:**
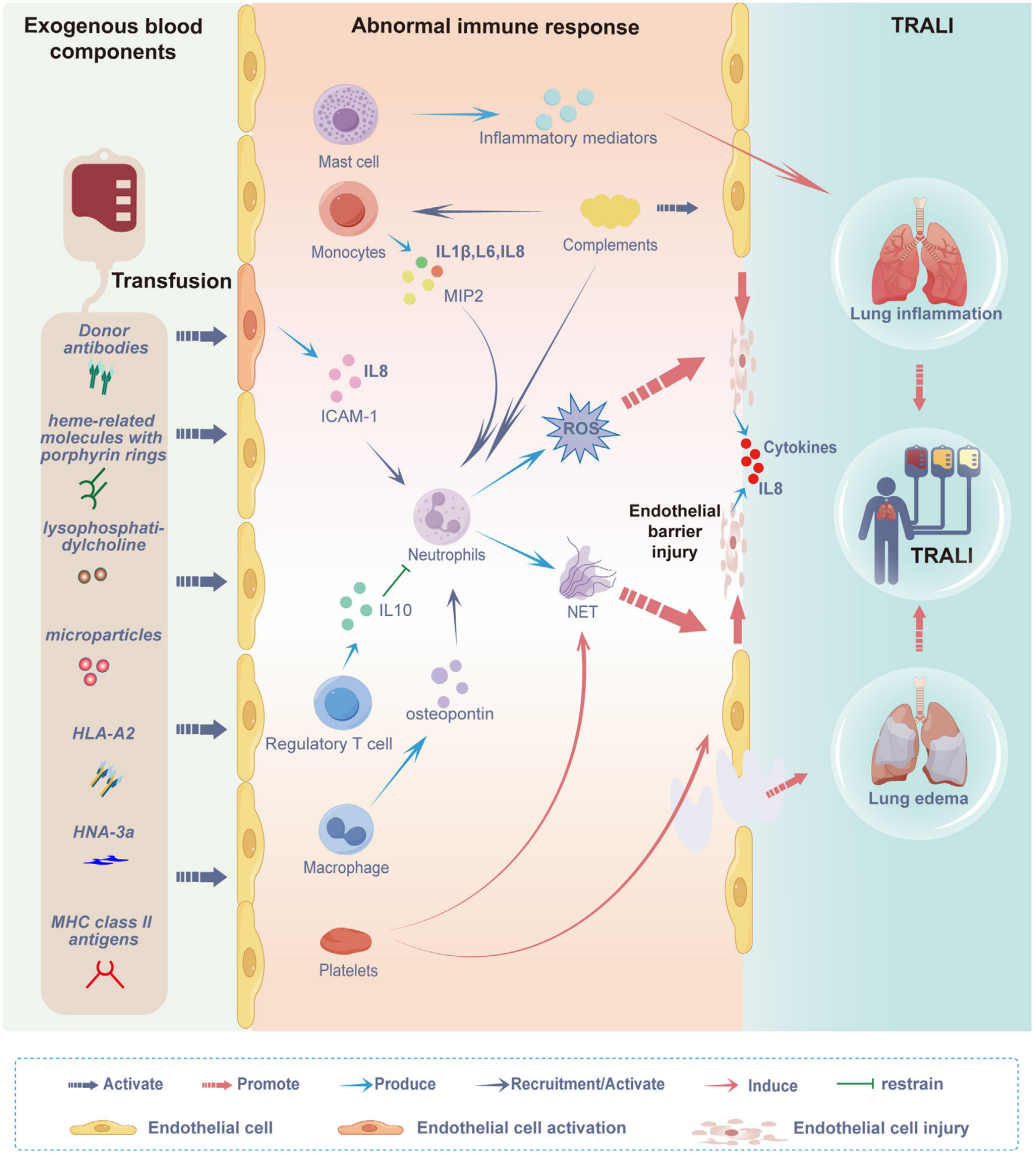
Cellular mechanisms underlying TRALI. Upon transfusion, antibodies and biologically active materials in blood products enter the circulation of the recipients. These components activate the immune system, leading to pulmonary inflammation and edema, which ultimately results in TRALI. The primary TRALI mechanism involves an abnormal immune response to exogenous blood components.

Animal models provide a foundation for investigating the cellular mechanisms of TRALI. Accumulated evidence from animal studies highlights the central role of immune mechanisms in this condition. Recent perspectives categorize TRALI as an immune‐mediated disorder^[^ ^12^ ^]^ triggered by an immune response to exogenous blood products (**Figure** **4**).

### 4.1 Neutrophils

Neutrophils, or polymorphonuclear neutrophils (PMNs), are crucial for the immune response. PMNs are abundantly present in the lungs of autopsied patients.^[^ ^66^ ^]^ Donor antibodies interacting with recipient PMNs activate and trap them in the lungs.^[^ ^67^ ^]^ Blood product components, including heme‐related molecules with porphyrin rings,^[^ ^68^ ^]^ lysophosphatidylcholine,^[^ ^69^ ^]^ microparticles,^[^ ^70^ ^]^ antibodies to HLA‐A2,^[^ ^71^ ^]^ HNA‐3a,^[^ ^46^, ^72^ ^]^ and major histocompatibility complex (MHC) class II antigens,^[^ ^45b^ ^]^ directly activate PMNs, exacerbating TRALI. Activated PMNs produce reactive oxygen species (ROS), damaging the endothelium in murine models.^[^ ^45^, ^56^, ^73^ ^]^ In addition, PMNs can capture and kill pathogens in the lungs using neutrophil extracellular traps (NETs). Excessive NETs cause severe tissue damage and inflammation.^[^ ^74^ ^]^ NETs are detectable in the lungs and plasma of patients with TRALI,^[^ ^56f,g^ ^]^ potentially forming during RBC storage.^[^ ^75^ ^]^ Microparticles in blood products^[^ ^76^ ^]^ and activated platelets^[^ ^56f^ ^]^ can trigger NET formation. NETs interact with immune components, contributing to TRALI pathobiology^[^ ^77^ ^]^ and mediating complement‐driven TRALI.^[^ ^56a^ ^]^ Notably, disulfiram protects against TRALI in mice by reducing NET formation.^[^ ^78^ ^]^ Moreover, antibody stimulation of PMNs induces heparin‐binding protein (HBP) release, contributing to TRALI.^[^ ^79^ ^]^ Antibody‐activated PMN‐mediated TRALI induction is Fc gamma receptor‐dependent.^[^ ^56^, ^80^ ^]^ However, autopsies of patients deceased owing to TRALI fatalities revealed no PMN infiltration in the alveoli,^[^ ^81^ ^]^ and TRALI occurred in one patient with neutropenia.^[^ ^82^ ^]^ In an antibody‐mediated TRALI mouse model, macrophages, not neutrophils, activated the complement system and generated C5a,^[^ ^56h^ ^]^ suggesting that PMNs are important, but not exclusive, TRALI pathogenesis contributors. Moreover, while neutrophil depletion reduces severity, it does not prevent TRALI in mice, positioning endothelial cells (ECs) as potential primary initiators.^[^ ^83^ ^]^

### 4.2 Endothelial Cells

ECs modulate the immune system, impacting immune cell function,^[^ ^84^ ^]^ and their dysfunction is a mechanism underlying ARDS development.^[^ ^85^ ^]^ EC necroptosis upon RBC transfusion releases receptor‐interacting protein kinase 3 (RIPK3) and high mobility group box 1 (HMGB1), aggravating lung inflammation via the receptor for advanced glycation end products (RAGE), confirmed by increased RIPK3 levels in patients with transfusion.^[^ ^53^, ^54^ ^]^ ECs could be TRALI‐initiating factors. The first hit activates vascular ECs, inducing cytokine release and increased intercellular adhesion molecule‐1 (ICAM‐1) expression, attracting and stimulating neutrophil adherence.^[^ ^67^, ^73^, ^86^ ^]^ After a blood transfusion, anti‐HNA‐3a antibodies trigger NOX‐dependent ROS release and increased EC permeability, exacerbating TRALI, with PMNs worsening this effect.^[^ ^83^ ^]^ Anti‐MHC class I antibody‐mediated ECs activate complement and attract monocytes/macrophages, leading to TRALI.^[^ ^56h^ ^]^ Vascular endothelial growth factor is released during platelet storage and acts as a permeability factor absorbed by the pulmonary vasculature, causing vascular injury and leakage.^[^ ^87^ ^]^ LPS and anti‐HLA class I antibodies synergistically enhance inflammatory responses in ECs via TLR4 and ICAM‐1.^[^ ^88^ ^]^ rIL‐35 prevents murine TRALI by inhibiting EC activation.^[^ ^89^ ^]^

### 4.3 Monocytes

Monocytes, key players in immune regulation, engulf pathogens, produce inflammatory mediators, and connect innate and adaptive immunity. Moreover, monocytes are established key players in HLA class II antibody‐induced TRALI, activating neutrophils and disrupting endothelial barriers, leading to pulmonary edema.^[^ ^90^ ^]^ Monocyte antigen binding to HLA antibodies releases inflammatory mediators, activating neutrophils and producing ROS, thereby promoting TRALI. Experimental evidence has confirmed that peripheral monocytes release MIP2, recruiting neutrophils to the lungs and causing injury via ROS.^[^ ^56e^ ^]^ Sakagawa et al. described that anti‐HLA class II antibodies activate monocytes via FcγR, leading to IL1β and IL6 production, chemotaxis, and increased neutrophil ROS release.^[^ ^91^ ^]^ Monocyte depletion in mice using gadolinium chloride prevented MIP2 production, neutrophil recruitment, and TRALI, while replenishing monocytes restored them.^[^ ^92^ ^]^

### 4.4 Regulatory T Cells

Regulatory T cells (Tregs), classified as natural and inducible Tregs (nTregs and iTregs, respectively), maintain immune homeostasis by suppressing excessive immune responses through cytokine suppression and metabolic disruption.^[^ ^93^ ^]^ nTregs are CD4+CD25+Foxp3+ cells, while iTregs include subtypes such as Tr1 and Th3, secreting IL10, or TGFβ. Foxp3‐expressing Tregs are crucial for immune tolerance and could be expanded *ex vivo* for therapy.^[^ ^94^ ^]^

Tregs rely on BLT1 for alveolar recruitment, supporting ALI resolution.^[^ ^95^ ^]^ In LPS‐mediated ALI, Tregs reduce neutrophil recruitment and activation via IL10 production.^[^ ^96^ ^]^ Tregs are critical for combating TRALI. CD4+CD25+Foxp3+ Tregs reportedly aggregate in the bronchoalveolar lavage fluid (BALF) of mice and patients with ALI, alleviating lung injury by mitigating inflammation.^[^ ^97^ ^]^ CD4+CD25+Foxp3+ Tregs resist antibody‐dependent mouse TRALI by producing IL10.^[^ ^56c^ ^]^ Expanding Tregs in vivo with IL2/IL2c increases IL10 and decreases IL17A levels, preventing antibody‐mediated TRALI.^[^ ^57^ ^]^ Moreover, subtypes such as Tr1 and iTR+35 cells display TRALI preventive potential, with rIL‐35 inhibiting EC activation in a mouse model.^[^ ^89^ ^]^

### 4.5 Macrophages

Macrophages are significant pathogenic players with diverse roles in health and disease.^[^ ^98^ ^]^ In antibody‐mediated murine TRALI models, macrophages are involved in C5a‐mediated ROS production, damaging the pulmonary endothelium.^[^ ^56h^ ^]^ They secrete osteopontin, localized in the lungs and attracting PMNs, leading to further endothelial damage via PMN‐ROS production.^[^ ^59a^ ^]^ M1‐polarized alveolar macrophages exacerbate TRALI, while α1‐antitrypsin can mitigate lung injury by reducing IL6 production and macrophage polarization.^[^ ^99^ ^]^ Targeting macrophage functions with C5a inhibitors, anti‐osteopontin antibodies, or α1‐antitrypsin treatment presents promising therapeutic strategies.^[^ ^100^ ^]^

### 4.6 Platelets

Platelets drive pulmonary and systemic coagulopathy in a TRALI model, mediated by lysophosphatidylcholines, which accumulate during storage.^[^ ^101^ ^]^ Hechler et al. reported that platelets prevent severe alveolar hemorrhages in TRALI but are not essential for the initiation of TRALI.^[^ ^56d^ ^]^ Platelets accumulate in the lung microvasculature, and their activation exacerbates TRALI, potentially through interactions with PMN.^[^ ^102^ ^]^ Platelet‐induced NETs accelerate TRALI.^[^ ^56f^ ^]^ Depleting platelets and aspirin treatment can protect mice with TRALI.^[^ ^30a^ ^]^ Moreover, platelets lead to soluble CD40 ligand (sCD40L) accumulation in the stored plasma, potentially contributing to TRALI.^[^ ^103^ ^]^ Stored platelets cause TRALI by transferring ceramides to ECs via extracellular vesicles.^[^ ^104^ ^]^ UVB light is used on platelet transfusion products to prevent alloimmunization and reduce pathogens, which might contribute to TRALI.^[^ ^105^ ^]^ In addition, platelets binding to chromatin within NETs activate platelets via glycoprotein (gp) IIb/IIIa receptors, releasing thromboxane A2 (TXA2), thereby enhancing neutrophil ROS release.^[^ ^106^ ^]^ Platelet FcγRIIA/CD32A activation exacerbates antibody‐mediated TRALI through enhanced serotonin release.^[^ ^107^ ^]^

### 4.7 Complement System

The complement system bolsters immune defense mechanisms; however, its dysregulation can cause inflammation and tissue damage.^[^ ^108^ ^]^ It is activated via C3 and C5 convertases, cleaving C3 into C3a and C3b as well as C5 into C5a and C5b, respectively. This process stimulates ECs, enhances C3b‐related phagocytosis, and forms the membrane attack complex for pathogen elimination.^[^ ^109^ ^]^ The complement system is involved in TRALI development and its activation is observed in the blood of patients with TRALI.^[^ ^56^, ^110^ ^]^ Increased C3d levels in patients in the ICU receiving plasma from multiparous donors correlate with lung injury, implicating the complement system in antibody‐mediated TRALI.^[^ ^111^ ^]^ Furthermore, studies using in vivo mouse models demonstrated that complement activation induces TRALI pathogenesis.^[^ ^56^, ^58^ ^]^ Anti‐CD36 antibodies can trigger TRALI, mitigated by C5 inhibitors, indicating a complement‐mediated mechanism.^[^ ^112^ ^]^ While C5a affects the anti‐MHC class I antibody‐induced TRALI model.^[^ ^56h^ ^]^ its role is not essential, as demonstrated in C5a receptor‐deficient mice.^[^ ^56j^ ^]^ The complement system might function synergistically with other immune components, affecting myeloid cells such as monocytes, macrophages, and neutrophils, which express the C5a receptor and contribute to TRALI.^[^ ^113^ ^]^ In addition, complement activation facilitates neutrophil functions and fragment crystallizable region‐mediated processes in macrophages, contributing to TRALI.^[^ ^56^, ^114^ ^]^ Cleary et al. confirmed that complement depletion via cobra venom factor protects mice from TRALI, suggesting that targeting complement activation could treat or prevent TRALI.^[^ ^110b^ ^]^

### 4.8 Mast Cells

Mast cells are central to the pulmonary immune system and enhance the immune response against pathogens.^[^ ^115^ ^]^ Although primarily associated with allergic reactions,^[^ ^116^ ^]^ mast cells also exhibit immunomodulatory effects.^[^ ^115^, ^117^ ^]^ Recent studies suggest that mast cells are involved in ARDS/ALI pathogenesis,^[^ ^118^ ^]^ and their stabilization could contribute to ameliorating acute lung injury.^[^ ^119^ ^]^ MHC‐I‐involved interactions might influence mast cell activation, suggesting their role in antibody‐mediated TRALI pathogenesis.^[^ ^120^ ^]^ Substances produced during lung injury, including thrombin, can trigger or enhance mast cell activation.^[^ ^121^ ^]^ Our previous study demonstrated that mast cells develop complex inflammatory profiles when exposed to exosomes from packed RBCs^[^ ^14^ ^]^ and confirmed their involvement in MHC‐I‐related TRALI,^[^ ^122^ ^]^ indicating their role in post‐transfusion immune responses.

### 4.9 Interaction Among Immune System Components in TRALI

Various immune system components are interconnected and synergistically contribute to TRALI pathogenesis. ECs are activated under the first hit, attracting and stimulating PMN.^[^ ^67^, ^73^, ^86^ ^]^ After transfusion, PMNs promote EC activation,^[^ ^83^ ^]^ thereby inducing complement cascades and attracting monocytes and macrophages to the lungs.^[^ ^56h^ ^]^ Antibodies could also activate PMNs^[^ ^71^, ^83^ ^]^ and monocytes,^[^ ^90a–c^ ^]^ leading to EC dysfunction. Activated monocytes increase neutrophil ROS release.^[^ ^91^ ^]^ Macrophages secrete osteopontin, attracting PMNs.^[^ ^59a^ ^]^ Stored platelets transfer ceramides to the ECs via extracellular vesicles.^[^ ^104^ ^]^ They can also activate PMNs and promote NET formation, thereby aggravating tissue damage.^[^ ^106^ ^]^ Complement‐affecting monocytes, macrophages, and PMNs contribute to TRALI.^[^ ^56^, ^113^, ^114^ ^]^ These interactions demonstrate that the synergistic interplay of multiple immune system components contributes to TRALI severity and disease progression.

## 5 Signaling Pathways

**Figure 5:**
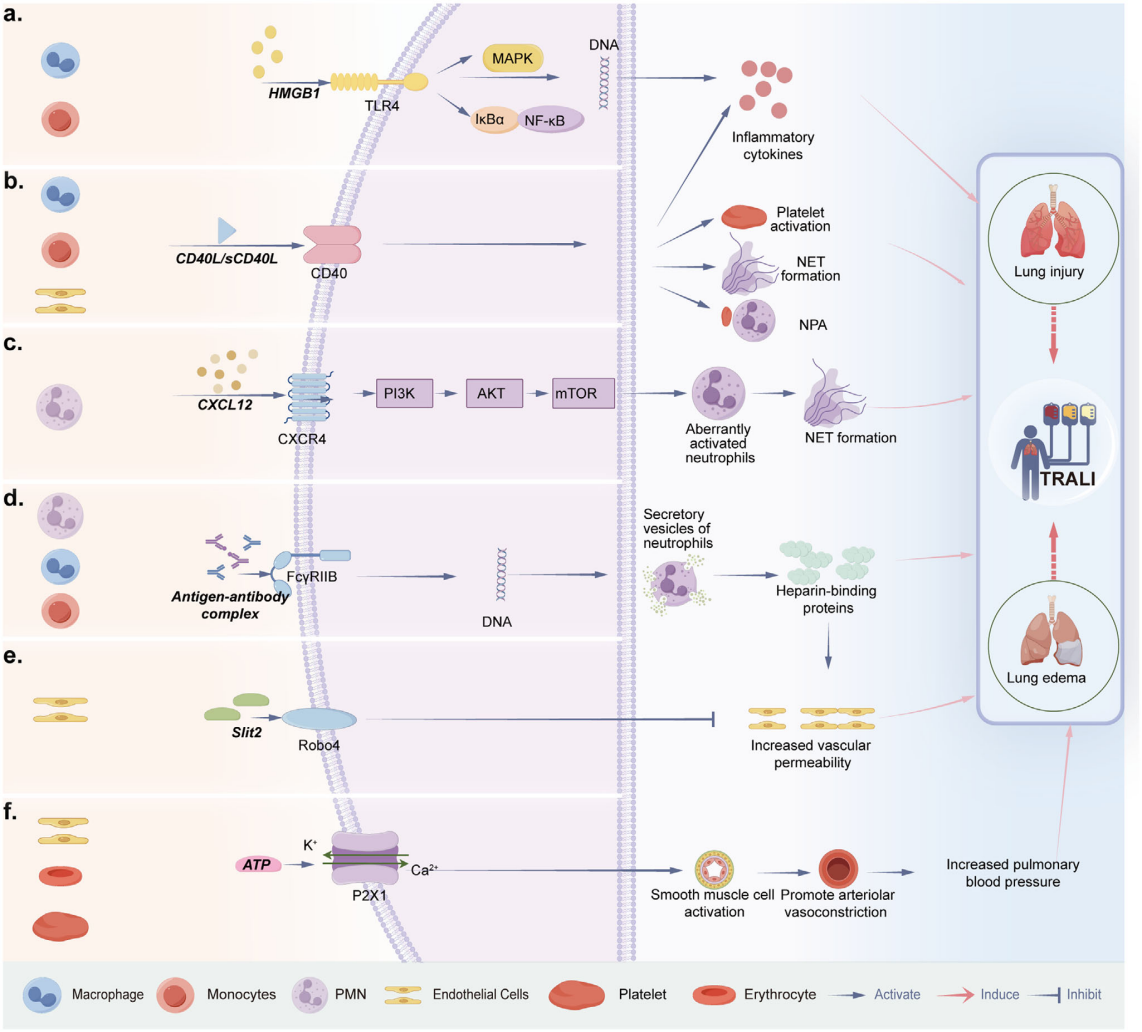
Signaling pathways in TRALI. Critical signaling pathways involved in the pathogenesis of TRALI. Left panels: Cells express those potential signaling pathways. a) HMGB1/RIP3 signaling pathway, b) sCD40L/CD40/CD40L signaling pathway, c) CXCR4/PI3K/Akt/mTOR signaling pathway, d) FcγRIIa/HBP signaling pathway, e) Slit2/Robo4 signaling pathway, and f) the ATP‐gated P2RX1 ion channel pathway.

The molecular mechanisms driving TRALI are intricately linked to key signaling pathways, which are essential for understanding the disease and developing effective treatments. TRALI involves complex inflammatory processes with several critical signaling pathways, including HMGB1/RIP3, sCD40L/CD40/CD40L, CXCR4/PI3K/Akt/mTOR, FcγRIIa/HBP, Slit2/Robo4, and the ATP‐gated P2RX1 ion channel pathway (**Figure** **5**).

### 5.1 Pro‐Inflammatory Signaling Pathways

#### 5.1.1 HMGB1/ RIP3 Signaling Pathway

HMGB1, a nuclear protein, and RIP3, an intracellular kinase, are key for inflammation. HMGB1 is released by activated monocytes and macrophages or necrotic cells, contributing to diseases such as ALI and chronic obstructive pulmonary disease (COPD).^[^ ^123^ ^]^ RIP3 induces cytokine production in immune cells.^[^ ^124^ ^]^ RBC transfusion triggers pulmonary endothelial necrosis, upregulating RAGE, which^[^ ^125^ ^]^ leads to HMGB1 and RIP3 release, exacerbating lung inflammation.^[^ ^126^ ^]^ HMGB1 and RIP3 activate the TLR4/NF‐κB/MAPK pathway, thereby promoting inflammation in rat lungs.^[^ ^15^ ^]^ Moreover, at increased levels, they serve as potential biomarkers for early TRALI diagnosis.^[^ ^15^ ^]^

#### 5.1.2 sCD40L/CD40/CD40L Signaling Pathway

CD40 is a type I transmembrane protein on endothelial and epithelial cells, monocytes, and macrophages.^[^ ^127^ ^]^ Its ligand, platelet‐derived CD40L, exists in soluble (sCD40L) and cell‐associated forms in transfused blood.^[^ ^128^ ^]^ The CD40/CD40L interaction displays a pro‐inflammatory role and is essential for various immune functions.^[^ ^129^ ^]^ sCD40L activates macrophages, inducing pro‐inflammatory cytokine production.^[^ ^130^ ^]^ Moreover, its association with platelets is significant in transfusion‐related complications,^[^ ^128^, ^131^ ^]^ and its levels correlate with platelet concentrate storage time.^[^ ^131^, ^132^ ^]^ In stored blood products, sCD40L can activate PMNs via CD40, leading to endothelial damage and potential TRALI in vitro.^[^ ^40^ ^]^ Notably, although treatment with ciglitazone can inhibit platelet CD40L expression, it does not affect TRALI mice.^[^ ^133^ ^]^ However, anti‐CD40L injections can reduce pulmonary edema and neutrophil‐platelet communication in TRALI mice, yielding fewer neutrophil‐platelet aggregates and reduced NET production.^[^ ^106^ ^]^ These data indicate that CD40L affects not only platelets but also other immune components in TRALI.

### 5.2 Neutrophil‐Related Signaling Pathways

#### 5.2.1 CXCR4/PI3K/Akt/ mTOR Signaling Pathway

CXCR4, a chemokine receptor on neutrophils, is activated by CXCL12, playing a key role in retaining PMNs in the bone marrow and preventing senescence.^[^ ^134^ ^]^ Proper neutrophil lifespan and activity regulation could prevent destructive hyperinflammatory states in target tissues, such as the lungs.^[^ ^135^ ^]^ CXCR4 activation triggers the PI3K/Akt/mTOR pathway.^[^ ^136^ ^]^ In TRALI models, inhibiting this pathway induces an aged PMN phenotype, reducing migration, granule content, and NET production, thereby improving survival rates.^[^ ^56^, ^135^, ^137^ ^]^

#### 5.2.2 FcγRIIa/ HBP Signaling Pathway

HBP is stored in neutrophil granules and induces endothelial cytoskeletal rearrangement, leading to cell barrier breakdown and increased macromolecular efflux.^[^ ^138^ ^]^ HBP regulates monocyte accumulation in the lung during acute inflammation,^[^ ^139^ ^]^ contributing to conditions such as sepsis^[^ ^140^ ^]^ and ARDS.^[^ ^141^ ^]^ Moreover, HBP induces ALI and vascular leakage via the TGF‐β/SMAD2/3 pathway.^[^ ^142^ ^]^ Fc gamma receptors (FcγRs), including FcγRIIa, are expressed on immune cells and mediate IgG antibody functions.^[^ ^143^ ^]^ FcγR–/– mice are resistant to MHC I antibody‐mediated lung injury, highlighting the impact of Fcγ receptors on neutrophils in TRALI pathogenesis.^[^ ^56j^ ^]^ In addition, PMN stimulation with human antibodies results in significant HBP release, mediated by FcγRIIIb and FcγRIIa,^[^ ^79^, ^91^ ^]^ suggesting that the FcγRIIa/HBP pathway is a major effector in TRALI.

### 5.3 Vascular and Endothelial Signaling Pathway

#### 5.3.1 ATP‐gated P2RX1 Ion Channel

Extracellular ATP, a purinergic signaling molecule, contributes to inflammatory diseases such as transplant rejection, autoimmune diseases, and bacterial infections.^[^ ^144^ ^]^ During ALI, ATP is released from the endothelial and immune cells, platelets, and stressed erythrocytes. The ATP‐gated ion channel P2RX1 receptor is involved in immune responses.^[^ ^145^ ^]^ LPS stimulation triggers ATP release, activating P2×1 receptors and leading to Ca^2+^ and K^+^ efflux.^[^ ^146^ ^]^ P2×1 prevents LPS‐induced endotoxemia by blocking neutrophil infiltration and activation,^[^ ^147^ ^]^ and might control pulmonary edema in TRALI.^[^ ^148^ ^]^ TRALI mice treated with the P2×1 receptor antagonist NF449 have been shown to develop lung edema, suggesting that P2RX1+ SMCs promote arteriolar vasoconstriction, increasing pulmonary blood pressure and favoring plasma leakage.^[^ ^148^, ^149^ ^]^

#### 5.3.2 Slit2/Robo4 Signaling Pathway

The Roundabout (*robo*) gene encodes a transmembrane receptor initially identified in *Drosophila*.^[^ ^150^ ^]^ Vertebrates express four Robo receptors, namely Robo1–4. The ligands for Robo receptors are Slit proteins, including Slit1–3, which are secreted and associated with the extracellular matrix.^[^ ^151^ ^]^ Slit2 specifically binds to Robo4.^[^ ^152^ ^]^

The semipermeable barrier function of the endothelium is critical in TRALI pathophysiology.^[^ ^83^, ^153^ ^]^ Slit‐Robo signaling promotes cell adhesion by stimulating the E‐cadherin‐β‐catenin interaction at the plasma membrane.^[^ ^154^ ^]^ Slit2‐mediated Robo4 activation inhibits the permeability of human lung microvascular ECs.^[^ ^152^, ^155^ ^]^ In TRALI studies, LPS‐induced PMN‐mediated TRALI significantly downregulates Slit2 and Robo4 expression levels in pulmonary microvascular endothelial cells and reduces the expression of the cell adhesion protein vascular endothelial‐cadherin,^[^ ^156^ ^]^ underscoring the importance of the Slit2/Robo4 signaling pathway in TRALI.

## 6 Potential Targeting Therapies

**Figure 6:**
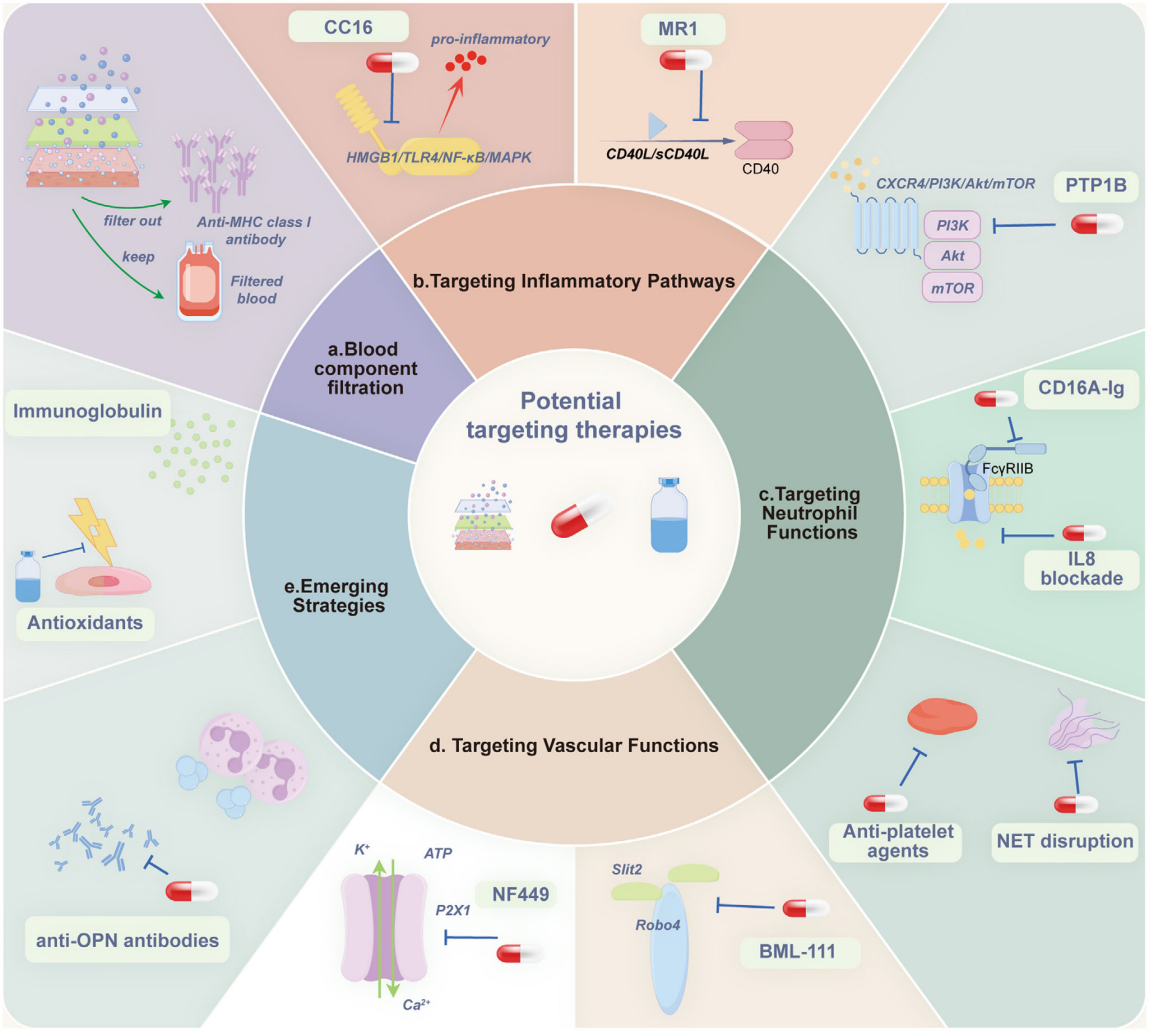
Potential TRALI‐targeting therapies. Potential targeting therapies for TRALI, including: a) Blood component filtration; b) Targeting inflammatory pathways, including HMGB1/RIP3 and CD40/CD40L signaling pathways; c) Targeting neutrophil functions, including the CXCR4/PI3K/Akt pathway, FcγRIIa signaling, IL‐8 and its receptors, neutrophil extracellular traps, and blood platelets; d) Targeting vascular functions, including the Slit2/Robo4 signaling pathway and ion channels; e) Emerging strategies, including reactive oxygen species (ROS), osteopontin, and intravenous immunoglobulin (IVIG).

The pathogenesis of TRALI is primarily triggered by immune responses to exogenous blood product components. Key pathogenic pathways, including inflammation, neutrophil activation, and endothelial dysfunction, play central roles in disease progression. Based on these molecular and pathway insights, various potential therapeutic strategies are being explored to mitigate the incidence and severity of TRALI. While these therapies are still in the preclinical research phase, targeting specific signaling pathways holds significant promise for developing effective treatments for TRALI (**Figure** **6**).

### 6.1 Blood Component Filtration

A strategy to reduce the risk of antibody‐mediated TRALI involves limiting patient exposure to plasma and avoiding unnecessary transfusions, particularly from female donors.^[^ ^37^, ^38^, ^90^, ^157^ ^]^ Experimental studies have explored antibody removal and biologically active materials from the blood components using leukocyte reduction filters with additional materials to adsorb antibodies and similar proteins. These filters inhibit anti‐MHC class I antibody‐mediated TRALI development in a two‐hit rat model. In contrast, unfiltered plasma containing antibodies induces TRALI in rats treated with LPS.^[^ ^158^ ^]^ Ongoing research continues to identify and validate additional biologically active factors in blood products that may contribute to abnormal immune responses. The integration of biomedical engineering and novel materials shows promise in removing these factors prior to transfusion, offering an effective strategy for preventing and treating TRALI.

### 6.2 Targeting Inflammatory Pathways

#### 6.2.1 Targeting the HMGB1/ RIP3 Signaling Pathway

CC16 is an anti‐inflammatory protein produced by club cells in the distal respiratory bronchioles. CC16 is a suggested biomarker for lung epithelial injury.^[^ ^159^ ^]^ It is involved in airway inflammatory diseases such as COPD and asthma.^[^ ^160^ ^]^ Increasing CC16 levels in the serum or lung tissues can inhibit inflammatory factors, thus reducing the inflammatory response.^[^ ^161^ ^]^ A previous study suggested CC16 as a specific biomarker for ARDS diagnosis and treatment.^[^ ^162^ ^]^ CC16 inhibits inflammatory cytokines via the HMGB1/TLR4/NF‐κB and MAPK/p38 pathways in ARDS, indicating its lung‐protective activity.^[^ ^163^ ^]^ Given that the same HMGB1/TLR4/NF‐κB/MAPK pathway is involved in TRALI,^[^ ^15^ ^]^ CC16 could potentially contribute to TRALI‐associated lung inflammation treatment by inhibiting this pathway.

#### 6.2.2 Targeting CD40/CD40L Signaling Pathway

sCD40L activates platelets, macrophages, and PMNs, inducing pro‐inflammatory cytokine production in TRALI.^[^ ^40^, ^128^, ^130^, ^131^ ^]^ Notably, anti‐CD40L antibodies have shown promising results in preclinical TRALI models by reducing pulmonary edema and neutrophil‐platelet aggregation, highlighting their potential as therapeutic agents for TRALI.^[^ ^106^ ^]^ This suggests that targeting the CD40/CD40L pathway could be an effective strategy to mitigate TRALI‐related inflammation. The monoclonal antibody MR1, which disrupts CD40‐CD40L interactions, has been demonstrated to effectively reduce lung injury,^[^ ^164^ ^]^ underscoring the potential of similar strategies in TRALI.

### 6.3 Targeting Neutrophil Functions

#### 6.3.1 Targeting the CXCR4/PI3K/Akt Signaling Pathway

CXCR4 inhibits neutrophil senescence via the PI3Kγ/AKT/mTOR pathway, leading to hyperinflammation and TRALI promotion, whereas CXCR4 inhibition reverses these effects.^[^ ^56b^ ^]^ Protein tyrosine phosphatase‐1B (PTP1B) regulates immune cell activation and polarization.^[^ ^165^ ^]^ PTP1B regulates B cell^[^ ^166^ ^]^ and macrophage polarization^[^ ^167^ ^]^ and increases IL10‐mediated TYK2/STAT3 anti‐inflammatory signaling.^[^ ^168^ ^]^ In a TRALI study, two PTP1B inhibitors (MSI‐1436 and DPM‐1003) induced PMN aging, attenuating lung injury, and decreasing mortality in murine models of TRALI and LPS‐induced endotoxemia.^[^ ^56^, ^169^ ^]^ This observation suggests that inhibitors alleviate TRALI by inhibiting PI3Kγ/AKT signaling downstream of CXCR4 and preventing NET formation in the lung tissue.^[^ ^56b^ ^]^

#### 6.3.2 Targeting FcγRIIa Signaling Pathway, IL8, and Its Receptors

FcγRIIa plays a critical role in the pathogenesis of TRALI by mediating immune responses to MHC I antibody‐induced lung injury. FcγR–/– mice show resistance to this type of injury, suggesting that FcγRIIa inhibitors may offer therapeutic benefits in TRALI.^[^ ^56^, ^143^ ^]^ Additionally, recombinant dimeric Fc receptor, CD16A‐Ig, has demonstrated protective effects in ARDS models,^[^ ^170^ ^]^ indicating its potential application in TRALI treatment. FcγRIIa also mediates the biological activities of anti‐IL‐8/IL‐8 complexes in vitro.^[^ ^171^ ^]^ Elevated IL‐8 levels in lung fluids are recognized as key prognostic indicators of ARDS outcomes.^[^ ^172^ ^]^ IL‐8 serves as a chemotactic factor, stimulating PMN chemotaxis and degranulation through CXCR1 and CXCR2 receptors.^[^ ^173^ ^]^ Increased IL‐8 levels have been observed in multiple TRALI cases, positioning it as a risk factor.^[^ ^27^, ^33^ ^]^ Blocking CXCR1/2 receptors may suppress PMN chemotaxis and degranulation, thereby counteracting TRALI. CXCR1/2 receptor antagonists, currently used to manage pulmonary inflammation and treating conditions such as COPD, asthma, and fibrotic lung diseases, may also exhibit potential efficacy against TRALI.^[^ ^174^ ^]^

#### 6.3.3 Targeting NETs and Blood Platelets

PMNs forming NETs are involved in TRALI.^[^ ^175^ ^]^ Platelets induce NETs in transfusion‐related ALI, contributing to lung endothelial injury. Therefore, targeting NET formation is a promising approach for ALI treatment.^[^ ^56f^ ^]^ Inhibiting platelet activation with aspirin or glycoprotein IIb/IIIa inhibitors, or directly targeting NET components with histone‐blocking antibodies and DNase1, can protect TRALI mice.^[^ ^30^, ^56^ ^]^ However, aspirin therapies remain controversial. Aspirin inefficacy in the treatment of critically ill patients with TRALI suggests that treatments effective in mouse models require further validation for human use.^[^ ^176^ ^]^ In addition, targeting neutrophil and platelet coupling and activation in TRALI is complicated by the risk of bleeding, as bleeding often necessitates blood transfusions.^[^ ^102^ ^]^

### 6.4 Targeting Vascular Functions

#### 6.4.1 Targeting the Slit2/Robo4 Signaling Pathway

BML‐111 is a lipoxin A4 receptor agonist with significant anti‐inflammatory properties, attenuating lung injury by affecting multiple cell types, including macrophages, epithelial cells, and ECs.^[^ ^177^ ^]^ BML‐111 activates the Slit2/Robo4 axis, reducing inflammatory markers, such as tumor necrosis factor α, IL6, and IL1β, in an LPS‐induced ALI model, thereby improving lung injury.^[^ ^178^ ^]^ The Slit2‐Robo4 pathway stabilizes vascular ECs and reduces their permeability.^[^ ^179^ ^]^ In TRALI mice, treatment with an active Slit2 fragment Slit2‐N increases Slit2/Robo4 and VE‐cadherin expression, protecting pulmonary microvascular ECs from neutrophil‐mediated hyperpermeability,^[^ ^156b^ ^]^ suggesting new TRALI therapies.

#### 6.4.2 Targeting the Ion Channels

During transfusion, various stimuli release ATP, activating the P2×1 ion channel. P2×1+ vascular smooth muscle cells promote arteriolar vasoconstriction and contribute to interstitial lung edema, a key process in TRALI.^[^ ^148^ ^]^ NF449, a selective P2RX1 antagonist, reduces TRALI severity in mice by decreasing lung periarteriolar interstitial edema. Maximum TRALI feature reduction requires treatment with NF449 before LPS sensitization and anti‐MHC I mAb administration.^[^ ^148^, ^180^ ^]^ TRPC6, another calcium channel expressed by smooth muscle cells, positively controls vasoconstriction, pulmonary arterial hypertension, and edema formation.^[^ ^181^ ^]^ The TRPC6 inhibitor SAR7334 reduces protein accumulation in BALs and periarteriolar edema in TRALI.^[^ ^181a^ ^]^

### 6.5 Emerging Strategies

PMN‐generated ROS play a significant role in the pathogenesis of TRALI, with studies showing that PMN depletion or inhibition of ROS production provides substantial protection against severe TRALI.^[^ ^56^, ^73^, ^83^ ^]^ Antioxidants, including high‐dose vitamins or small‐molecule oxidation inhibitors, have shown promise as therapeutic options for TRALI by mitigating ROS‐induced damage.^[^ ^182^ ^]^

Intravenous immunoglobulin (IVIG), derived from human blood, is effective in treating immune disorders, including immune hematologic conditions.^[^ ^183^ ^]^ When administered prophylactically before TRALI induction, IVIG prevents systemic shock, lung injury, and mortality in antibody‐mediated TRALI by inhibiting ROS generation.^[^ ^184^ ^]^ However, IVIG may also carry the risk of inducing TRALI due to anti‐leukocyte antibodies, as demonstrated by a case where TRALI occurred after IVIG infusion, with granulocyte‐associated IgG detected in the patient's blood.^[^ ^41a^ ^]^

Osteopontin is a pro‐inflammatory cytokine regulating the adhesion and migration of immune cells, including PMNs. It is highly expressed in various lung diseases.^[^ ^185^ ^]^ Osteopontin (*Opn*)‐knockout mice exhibit resistance to antibody‐mediated PMN‐dependent TRALI, suggesting that blocking osteopontin with anti‐osteopontin antibodies may be a potential therapeutic intervention for TRALI.^[^ ^59a^ ^]^

## 7 Summary and Perspectives

**Figure 7:**
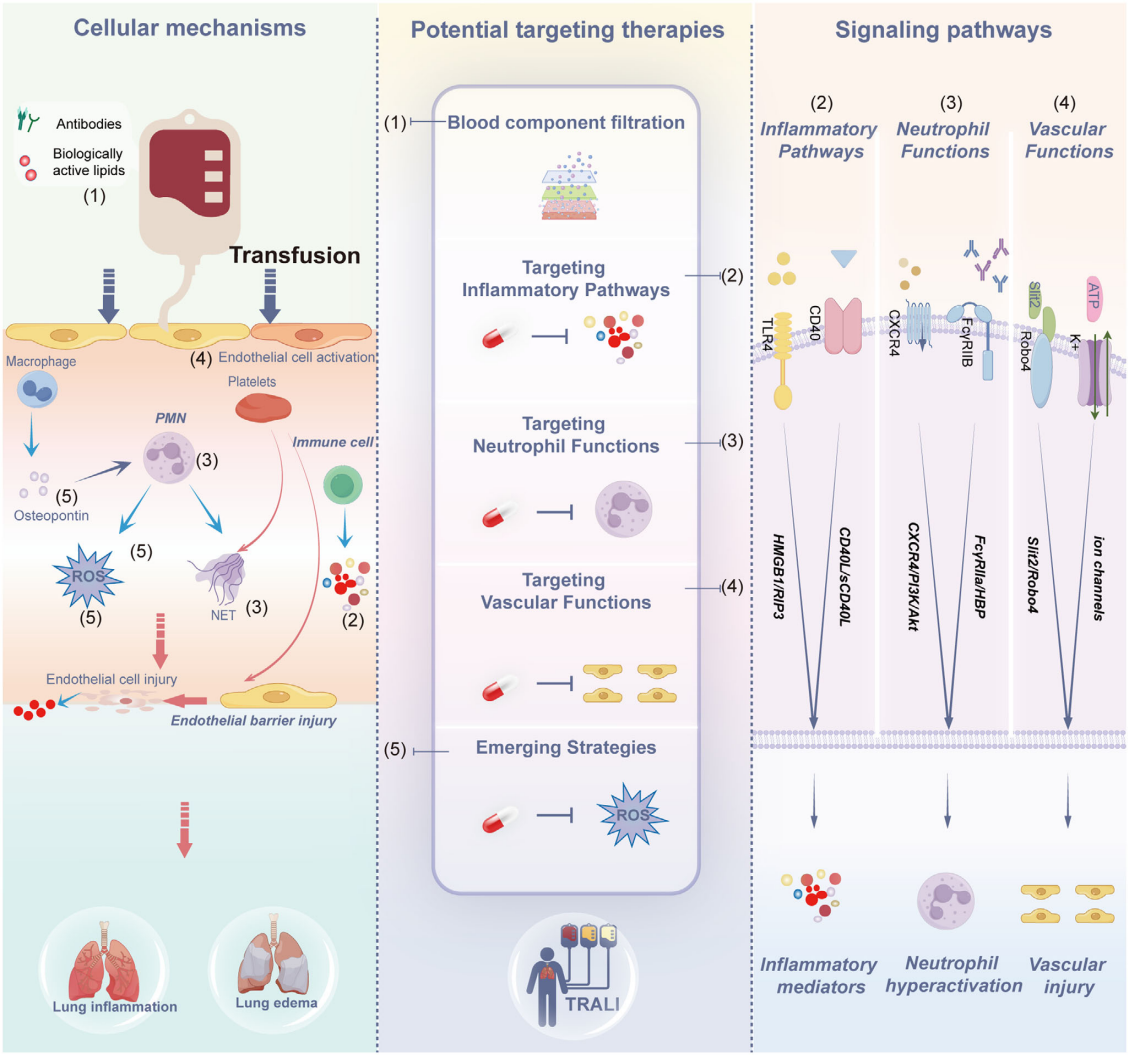
Cellular mechanisms, signaling pathways, and potential therapeutic targets in TRALI. The pathogenesis involves immune responses to immunogenic blood product components, with key pathways including inflammation, neutrophil activation, and endothelial dysfunction. Based on these molecular and pathway insights, therapeutic strategies focus on removing immunogenic components of exogenous blood products, modulating inflammation, and targeting neutrophils and endothelial cells. Emerging therapies, such as those targeting reactive oxygen species (ROS), intravenous immunoglobulin (IVIG), and osteopontin, are in preclinical stages.

This review provides a comprehensive analysis of the clinical characteristics, pathogenetic hypotheses, animal models, cellular mechanisms, signaling pathways, and potential therapeutic targets for TRALI. Despite its underestimated incidence and mortality, particularly in critically ill patients, providing a comprehensive framework to enhance understanding and guide future research for better diagnosis and treatment. TRALI remains a challenging clinical condition due to the lack of specific diagnostic markers and targeted therapies. The pathogenesis is driven complex immune mechanisms, primarily triggered by immune responses to exogenous blood product components. Key pathogenic pathways include inflammation, neutrophil activation, and endothelial dysfunction. Building on these molecular and pathway insights, therapeutic strategies have emerged focusing on removing immunogenic blood product components, modulating inflammatory responses, and targeting neutrophils and endothelial cell functions (**Figure** **7**). Emerging therapies, such as those targeting ROS, osteopontin, and IVIG, are currently in preclinical development (**Table** **1**).

**Table 1:** Cellular mechanisms, signaling pathways, and potential therapeutic targets in TRALI.

Cellular Mechanisms	Signaling Pathways	Potential Targeting Therapies	Supporting Literature/Studies
Exogenous Blood Products	/	Blood Component Filtration	^[^ 37, 38, 90,^157^ ^]^
Immune cell type	Pro‐inflammatory signaling pathways	Targeting inflammatory pathways	
Monocytes/ Macrophages/ Endothelial cells	HMGB1/RIP3 signaling pathway	Targeting HMGB1/RIP3 signaling pathway	[15]
Monocytes/ Macrophages/ Endothelial cells	sCD40L/CD40/CD40L signaling pathway	Targeting CD40/CD40L signaling pathway	[164]
Immune cell type	Neutrophil‐related signaling pathways	Targeting neutrophil functions	
Neutrophils	CXCR4/PI3K/Akt/mTOR signaling pathway	Targeting CXCR4/PI3K/Akt signaling pathway	[56, 169]
Neutrophils/Endothelial cells/ Monocytes	FcγRIIa/HBP signaling pathway	Targeting FcγRIIa signaling pathway, IL8, and its receptors	[170]
		Targeting NETs and blood platelets	[30, 56]
Immune cell type	Vascular and endothelial signaling pathways	Targeting vascular functions	
Endothelial cells/ Neutrophils/	ATP‐gated P2RX1 ion channel	Targeting the ion channels	[181a]
Endothelial cells/ Neutrophils/	Slit2/Robo4 signaling pathway	Targeting the Slit2/Robo4 signaling pathway	[156b]
		Emerging strategies	
		Targeting ROS Targeting osteopontin Intravenous immunoglobulin	^[^ ^182^ ^]^^[^ ^184^ ^]^^[^ ^59a^ ^]^

The clinical overview highlights the lack of specific diagnostic markers in TRALI. Comparative sequencing of pre‐ and post‐transfusion samples from high‐risk patients, including those with and without TRALI, could help to identify predictive factors. Given the unpredictable nature of TRALI incidence, prospective studies in high‐risk populations are essential to validate these markers and enhance diagnostic efficiency. The synthesis of hypotheses underscores the multi‐faceted nature of TRALI, which involves both antibody‐mediated and non‐antibody‐mediated pathways. Developing multi‐dimensional diagnostic tools that incorporate biomarkers such as antibody activity, complement activation, and cytokine levels could address this complexity and significantly improve early detection. Integrating omics technologies and artificial intelligence (AI)‐based approaches into future research could validate these markers and facilitate the development of an advanced multi‐dimensional diagnostic platform.

Mechanistic studies reveal that TRALI is primarily driven by abnormal immune responses to exogenous blood components. Identifying and removing immune‐activating factors, such as bioactive lipids and exosomes, could significantly reduce TRALI incidence. Advances in biomedical engineering could enable the development of novel materials or filtration technologies designed to selectively remove these substances from blood products pre‐transfusion, thereby minimizing the risk of abnormal immune responses. While PMNs, inflammation, and endothelial cells are primary contributors to the pathogenesis of TRALI, secondary components such as the complement system, platelets, and mast cells also play pivotal roles in amplifying the immune response. Given the complexity of the immune system, further validation and targeting of these components could aid in prevention and treatment.

Although animal models are invaluable for studying TRALI mechanisms, they often replicate only specific aspects of the condition. Biological factors identified from clinical samples should be validated in these models to confirm their roles in TRALI. Functional studies, such as gene knockout experiments or exogenous factor injection, can clarify causality. Future efforts should also integrate in‐vitro models and clinical cohorts to ensure translational relevance. Pathway studies based on animal models have revealed several therapeutic targets, but clinical validation remains a challenge due to TRALI's unpredictable incidence and the limitations of single‐center randomized controlled trials. Establishing standardized diagnostic criteria and management guidelines through multicenter collaborations is crucial. International consortia should focus on large‐scale clinical trials and data‐sharing initiatives to accelerate the development of effective diagnostic and therapeutic solutions. Additionally, the lack of a unified TRALI database hinders large‐scale studies, and establishing one could advance research, diagnosis, and treatment.

In conclusion, this review highlights that the primary cause of TRALI is an abnormal immune response to exogenous blood components. Therapeutic strategies, grounded in molecular and pathway insights, focus on the removal of immunogenic blood product components, modulation of inflammation, and targeting neutrophil and endothelial cell functions. Future research should prioritize the identification of reliable diagnostic biomarkers based on clinical sample sequence analysis, the development of targeted therapies, and the exploration of innovations in medical engineering, AI‐assisted technologies, multicenter studies, and novel materials, with validation through multicenter randomized trials. Addressing these challenges will be essential for improving patient outcomes and alleviating the burden of this severe condition.

## Conflict of Interest

The authors declare no conflict of interests.

## Author Contributions

Dr. Chunheng Mo is a co‐first author. X.B.F., F.G., and L.Z. wrote and finalized the manuscript; X.B.F. designed the study; X.B.F., F.S.X, X.C.Z., and C.H.M. revised the manuscript.
